# A model to assist psychiatric nurses in facilitating support of parents who are caring for an adolescent with a mental disorder

**DOI:** 10.4102/curationis.v49i1.2834

**Published:** 2026-02-19

**Authors:** Hendrietta Vorster-Mbontsi, Nompumelelo Ndlovu, Annie Temane

**Affiliations:** 1 Department of Nursing, Faculty of Health Sciences, University of Johannesburg, Johannesburg, South Africa

**Keywords:** adolescent, caring, facilitate, mental disorder, model, parents, psychiatric nurses, support

## Abstract

**Background:**

An estimated one in seven adolescents globally experiences a mental disorder, and parents, as primary caregivers, play an essential role in caring for their adolescent with a mental disorder. Consequently, parents face many challenges and require support from psychiatric nurses.

**Objectives:**

To describe and evaluate the development of a model for psychiatric nurses to facilitate support for parents who are caring for an adolescent with a mental disorder.

**Method:**

A theory-generative, qualitative, exploratory, descriptive and contextual study design was used to develop the model. The central concept of the model was derived from a previous study. The process entailed identifying the central concept and other essential criteria, classifying the central concepts and describing the relationships between them.

**Results:**

The central concept was identified as ‘facilitation of support’ for parents who are caring for an adolescent with a mental disorder. The identified and defined central concepts were placed into interrelated statements. The model to facilitate support for parents who are caring for an adolescent with a mental disorder was developed, described and evaluated. The model has not been implemented.

**Conclusion:**

The model provides facilitation of support for parents who are caring for an adolescent with a mental disorder. Additionally, the model empowers psychiatric nurses with the skills to facilitate support for parents.

**Contribution:**

The model contributes to the facilitation of support for parents who are caring for an adolescent with a mental disorder. This model will also contribute to the empowerment of psychiatric nurses.

## Introduction and background

Adolescence is a critical developmental period that begins at puberty and continues until the mid-1920s (eds. Backes & Bonnie 2019:n.p.). This stage is characterised by accelerated growth patterns, emotional and behavioural changes and psychological development (Uktamovna 2025:169). In recent years, mental disorders have become a leading cause of disability among adolescents worldwide, with an estimated one in seven 10–19-year-olds experiencing a mental disorder. This accounts for an estimated 15% of the global burden in this age group (World Health Organization 2025:n.p.), with depression, anxiety, attention-deficit hyperactivity disorder and dissocial disorder being the most common (Moyo et al. 2025:2). Mental health challenges in adolescents are primarily associated with poor academic performance, substance abuse, physical illness and behavioural struggles (Patel et al. 2007:1302).

Mental disorders disrupt the normal development of adolescents and hinder their participation in age-appropriate activities, such as school, and generally being independent (Herpertz-Dahlmann, Bühren & Remschmidt 2013:n.p.). This affects not only adolescents but also places a significant burden on their families, particularly their parents or primary caregivers (Phillips et al. 2023:5). Parents play a critical role and face many challenges in caring for an adolescent with a mental disorder. This can lead to parents suffering overwhelming distress, social isolation and burnout, negatively impacting their overall quality of life and life satisfaction while increasing the burden of care (Phillips et al. 2023:8). Despite their critical role, parental support is usually informal, unstructured and not integrated into standard mental healthcare (Weisenmuller & Hilton 2021:107). There is a significant gap between the need for parental support and the availability of evidence-based, structured interventions provided by mental healthcare professionals within clinical mental health settings (Weisenmuller & Hilton 2021:121–122).

The challenges that adolescents with mental illness experience extend to their parents (Boritz et al. 2021:579).

Caregiving parents of an adolescent who has a mental disorder deal with a variety of emotional, psychological and interpersonal barriers (Sarrió-Colas et al. 2022:45). Many parents experience burden along with shame, rejection or blame from family members, friends and the community, who stigmatise and isolate these adolescents (Bai et al. 2020:777; Boritz et al. 2021:579). Furthermore, parents of adolescents living with a mental disorder often struggle with negative emotions, such as profound guilt, worry and anxiety about whether someone else will be able to assume the caregiving role if they are no longer capable (Corcoran, Berry & Hill 2015:356; Klages, Usher & Jackson 2016:6).

Despite the development of various interventions, frameworks and strategies targeted at parents and adolescents – such as the Systematic Training for Effective Parenting (STEP) programme, family support programmes, family or individual therapy, psycho-educational programmes and support groups – no model currently exists to facilitate the mental health of parents caring for an adolescent with a mental disorder in South Africa, particularly one implemented by psychiatric nurses. The knowledge gap regarding the burden of mental disorders remains a challenge in sub-Saharan Africa (Jörns-Presentati et al. 2021:18). To address this gap, this study will develop, describe and evaluate a model for psychiatric nurses to facilitate the mental health of parents caring for an adolescent with a mental disorder.

Based on the lack of mental health services for parents caring for an adolescent with a mental disorder, the following research question arose: *What can be done to facilitate the mental health of parents caring for an adolescent with a mental disorder?*

The development of a model for psychiatric nurses to facilitate support for parents who are caring for an adolescent with a mental disorder will not only benefit the parents but will also empower psychiatric nurses by equipping them with the knowledge and skills to enhance their confidence in providing support to parents (Darawad et al. 2025:16–17). Furthermore, psychiatric nurses must facilitate this model as they are the first point of contact with the parents who are caring for an adolescent with a mental disorder.

Facilitating support for parents of adolescents with mental disorders is vital because parents often experience significant stress, uncertainty and emotional burden when caring for an adolescent with a mental disorder (Al Yahyaei et al. 2024:6). Improved support for parents enhances the parents’ ability to cope and contributes to a more positive family functioning (Lakhani et al. 2025:150). Furthermore, supported parents are better equipped to collaborate with healthcare professionals, which contributes to improved treatment adherence and overall outcomes for the adolescent.

## Research methods and design

### Study design

A theory-generative, qualitative, exploratory, descriptive and contextual research design (Chinn, Kramer & Sitzman 2022; Grove & Gray 2022:100) was employed to develop a theory. The study used the following three phases: empirical phase (Phase 1), concept analysis (Phase 2) and development (Phase 3).

#### Purpose of the study

This study aims to develop and describe a model for psychiatric nurses to facilitate support for parents who are caring for an adolescent with a mental disorder. The model is based on the authors’ research findings and the Theory of Health Promotion as used by the University of Johannesburg, South Africa (University of Johannesburg 2017:11; Vorster, Ntshingila & Poggenpoel 2021). Chinn and Kramer’s guidelines for theory description were used to describe the model (Chinn et al. 2022:162).

#### Phase 1: Empirical phase

In-depth phenomenological individual interviews with parents who are caring for an adolescent with a mental disorder were conducted.

**Population and sample:** The study’s population was parents of adolescents who were admitted to the psychiatric unit of a public hospital. Ten parents were purposively sampled to participate in this research study (Chukwuere, Sehularo & Manyedi 2022:2; Polit & Beck 2017:741). The inclusion criteria were one or both parents of an adolescent aged 14–19 years admitted to the psychiatric unit of a public hospital in Gauteng. Parents had to be able to communicate in English and have access to a cell phone or landline. Parents who did not meet the inclusion criteria were excluded from the sample.

**Data collection:** The first researcher conducted 10 in-depth telephonic interviews. These interviews were conducted in the participants’ homes, where they were most comfortable. Telephonic interviews were conducted owing to the national shutdown caused by the coronavirus disease 2019 outbreak. The participants were provided with all the information about the study, and participation was voluntary. Permission to audio-record the telephonic interviews was obtained from the participants. The autonomy of the participants was respected by allowing them to make an informed decision before signing an informed consent form. Their confidentiality was respected throughout the study, as their names were not exposed; codes were used to identify the participants. The participants were not under any obligation to participate in the research and could withdraw at any time. This was indicated in the participant’s information letter. The researcher asked the participants one central question: ‘What is it like to have an adolescent with a mental disorder?’ Each in-depth interview with the parents lasted between 45 and 60 minutes. Data were also collected in the form of field notes during the interviews. Only the researcher and the participants were present during the interviews. The researcher made field notes after each in-depth interview. The in-depth interviews were transcribed verbatim, incorporating both observational and field notes. Data saturation was reached at the sixth interview, and four additional interviews were conducted to confirm saturation.

**Data analysis:** The eight steps of Tesch’s thematic coding were used to analyse the collected data (Creswell 2014). The researcher captured the data using audio recordings and field notes. Recorded interviews were transcribed verbatim, which provided the database for analysis. The researcher closely examined the data to identify recurring themes, topics, ideas and patterns of meaning. The supervisor and co-supervisor also examined the transcribed interviews before handing them to an independent coder. The independent coder, experienced in qualitative research, analysed the data separately from the researcher. The researcher and independent coder then met for a consensus discussion on the data analysis findings. The researcher and independent coder agreed on the final themes. These findings were then recontextualised in the literature after the data analysis.

**Measures to ensure trustworthiness:** The four measures of trustworthiness in qualitative research, as outlined by Lincoln and Guba (1985), were applied: credibility, transferability, dependability and confirmability. Credibility was ensured through prolonged engagement, which involved staying in the field until data saturation was reached. The researcher conducted interviews with parents that lasted 45–60 minutes each and was in the field for a period of 2 months.

Each participant was interviewed only once, with no repeat interviews conducted. Triangulation was achieved by employing various data collection methods and peer debriefing, which involved engaging with experts to discuss and refine the research process. The researcher ensured dependability through an inquiry audit by providing the study’s documentation to external reviewers for scrutiny. The documentation included the study timeline, raw data and notes taken during data collection and analysis. The researcher provided a detailed description of the study, enabling readers to evaluate the adequacy of the analysis and the reasoning behind the conclusions. To enhance confirmability, the researcher employed an audit trail, maintained access to the raw data, shared interviews with experts in the field and held regular consultations with research supervisors.

Transferability was achieved by the researcher providing dense descriptions of the participants’ demographics and offering rich descriptions of the results, including direct quotations from the participants. Purposive sampling was used to enhance transferability by purposefully selecting participants with knowledge of the research problem.

**Ethical considerations** The researcher obtained permission to conduct the study from the University of Johannesburg’s Higher Degrees Committee (ref no.: HDC-01-64-2023) and Research Ethics Committee of the Faculty of Health Sciences (ref no.: REC-2111-2023). Permission to conduct the research was also obtained from the Chief Executive Officer and the Research Committee of the Psychiatric Hospital. The researcher adhered to the ethical principles of autonomy (Gray & Grove 2021:101), beneficence and non-maleficence (Polit & Beck 2017:139) and justice (Middleton 2020:1340). A detailed explanation of the study’s purpose was provided to the participants. All participants were informed of the risks and benefits of the study. Verbal and written consent were obtained from participants before the commencement of the interviews. The participants were informed of their right to withdraw from the study at any time without facing consequences. The participants’ right to privacy, confidentiality and anonymity were adhered to. Anonymity was ensured by using pseudonyms to represent the participants.

**Findings of the study:** Three themes emerged from the experiences of parents of adolescents who were admitted to the psychiatric unit of a public hospital. The findings revealed that parents experienced their lives being suspended between grief for the loss of what their adolescents had once been and anxiety about what the future might hold. The findings further indicated that when parents sought professional help, the healthcare system failed to provide them with adequate guidance, information and support. Ultimately, the parents experienced that their only remaining coping strategy was to pray for divine intervention (Vorster et al. 2021:55–56).

#### Phase 2: Concept analysis

This phase consists of two steps: concept identification and concept classification (Chinn et al. 2022:162). The researcher utilised her minor dissertation findings (Vorster et al. 2021) to assist in identifying the central concepts for this research study, which were then defined and classified.

**Step 1: Concept identification:** To identify concepts as building blocks for the model, the researcher employed an inductive theory-generative research design (Chinn et al. 2022:162). The research design and procedures are discussed later to explain how the concepts were identified.

**FIGURE 1:**
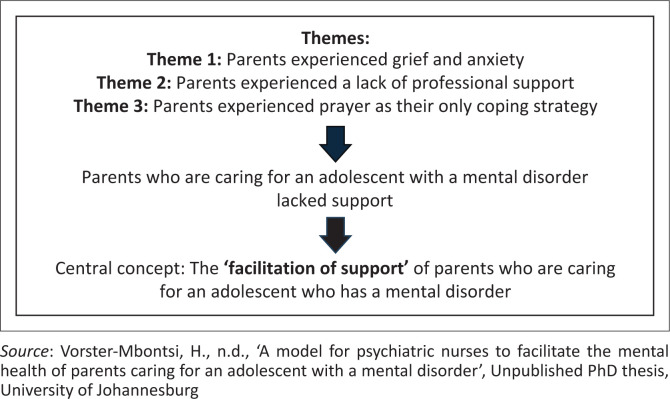
Identifying the central concept.

**Identification of the central concept:** Figure 1 illustrates the central concept identified through the analysis of the data collected in the researcher’s minor dissertation. The themes were used to identify the central concept. The central concept that emerged from the data was identified as the ‘facilitation of support’.

**Step 2: Definition and classification of the central concept:** Chinn et al. (2022:162) support the idea that defining the central concept gives it meaning. The definitions of concepts were performed both connotatively and denotatively, using dictionaries and available literature (Walker & Avant 2019:208). The essential attributes for defining the central concept were identified from the dictionary and subject-specific definitions. ‘Facilitation of support’ was defined based on these essential attributes (see Table 1). The essential attributes were further illustrated in a model case. Because of the unpublished nature of the definition, detailed elaboration is beyond the scope of this manuscript. The central concepts were classified using Dickoff, James and Wiedenbach’s (1968:425) survey list, considering the following: Who is the agent? Who is the recipient? What is the procedure? What are the dynamics? What is the context? And what is the outcome? The classification of the central concept is outlined below:

- The agent is the psychiatric nurse. The psychiatric nurse engaged with the parents who are caring for an adolescent with a mental disorder. To perform this activity, the agent must have internal resources, including skills, techniques, routines or standard-operating procedures (Dickoff et al. 1968:426). The agent must have external skills, including maintaining, supporting, developing and protecting (Dickoff et al. 1968:426). The agent is responsible for implementing the model to facilitate support for parents caring for an adolescent with a mental disorder.
- The recipients are the parents caring for an adolescent with a mental disorder. The parents benefited from the facilitation of support by the psychiatric nurses, which is a crucial part of mental health.
- The procedure involves using the model to facilitate support to parents as a vital part of mental health. The facilitation of support took place in three phases: the relationship phase, the working phase and the termination phase.
- The dynamics were identified from the experiences of parents of an adolescent with a mental disorder admitted to the psychiatric unit of a public hospital. The parents experienced grief, anxiety and lack of professional support, with prayer being their only coping strategy. They reported both positive and negative experiences, which reflected the dynamics relevant to the classification of the concept. The persistent grief necessitates a model that mentors and provides psychiatric nurses with training in validation, grief-sensitive and empathetic communication. The debilitating anxiety demands a model centred on education and shared responsibility. The profound lack of support, evidenced by parents relying solely on prayer for solace, is directly addressed by a model that formalises the psychiatric nurse’s role as approachable, proactive and a resource provider. Therefore, each component of the model is a deliberate intervention developed to mitigate these specific emotional and systemic challenges, transforming the psychiatric nurse’s role from a minor caregiver to the essential facilitator of parental resilience. These dynamics highlighted the need for psychiatric nurses to facilitate support for parents caring for an adolescent with a mental disorder.
- The context of this study refers to the setting in which the model will be implemented to facilitate support for parents caring for an adolescent with a mental disorder.
- The terminus represents the outcome, whereby the parents caring for an adolescent with a mental disorder experience support. This constitutes the end result of the activity.

**TABLE 1:** The essential attributes of ‘facilitation of support’.

Essential concept	Essential criteria
Facilitation	Assist
	Dynamic, interactive process
	Promotion of learning and growth
Support	Human interaction
	Providing guidance
	Material and immaterial resources

*Source:* Vorster-Mbontsi, H., n.d., ‘A model for psychiatric nurses to facilitate the mental health of parents caring for an adolescent with a mental disorder’, Unpublished PhD thesis, University of Johannesburg, Johannesburg

**Describing relationships between the concepts:** Relationship statements structurally describe concepts of the model that are correlated; it involves describing, explaining or predicting the relationships between these concepts (Chinn et al. 2022:153). Concepts are linked through a process that illustrates how the identified concepts and their characteristics meaningfully interrelate (Chinn et al. 2022:153). In this study, the relationship statements described the interconnections between the selected concepts, demonstrating how the essential concepts relate to one another. The relationship statements were formulated as outlined below. The psychiatric nurse facilitates support through a dynamic, interactive process with the parents who are caring for an adolescent with a mental disorder. The psychiatric nurse assists the parents by promoting learning and growth. They promote human interaction by displaying empathy and establishing a trusting, therapeutic relationship with parents who are caring for an adolescent with a mental disorder. They provide guidance through mobilising material and immaterial resources for parents who are caring for an adolescent with a mental disorder. Parents’ mental health is promoted through the facilitation of support by psychiatric nurses.

#### Phase 3: Development of a model to facilitate support for parents who are caring for an adolescent with a mental disorder

**FIGURE 2:**
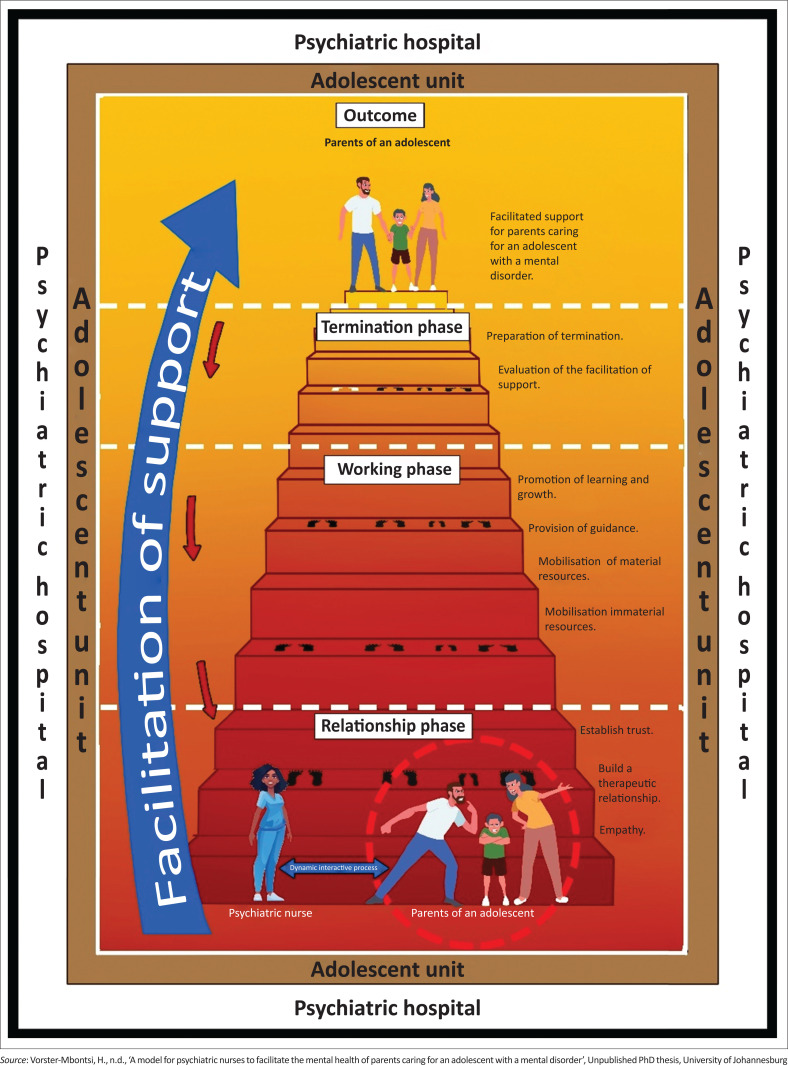
A model for psychiatric nurses to facilitate support for parents who are caring for an adolescent with a mental disorder.

**Description of the model:** The criteria for descriptive components of a model were applied in describing the model (Chinn et al. 2022:177–179). The criteria used for describing the model for psychiatric nurses to facilitate support for parents who are caring for an adolescent with a mental disorder included assumptions, purpose, conceptual definition, relationship statements and the structural description of the model. Figure 2 presents the model designed for psychiatric nurses to facilitate support for parents caring for an adolescent with a mental disorder.

**Structure of the model:** The structure of the model is discussed using the headings as outlined by Chinn et al. (2022:177–179), assumptions of the model, purpose of the model and process description of the model.

**Assumption of the model:** The assumptions of the model of this study were grounded in the Theory for Health Promotion in Nursing (University of Johannesburg 2017:5). The assumptions that underlie this model are reflected in the following statements (University of Johannesburg 2017:5): Parents who are caring for an adolescent with a mental disorder are viewed holistically and integrated into both the internal and external environments. The internal environment consists of the body, mind and spirit dimensions, while the external environment comprises the physical, social and spiritual dimensions.

**Purpose of the model:** The purpose of the model was to assist psychiatric nurses in facilitating support for parents who are caring for adolescents who have a mental disorder.

**Conceptual definition:** Facilitation of support refers to a dynamic and interactive process between the psychiatric nurse as a facilitator and the parents who are caring for an adolescent with a mental disorder. The psychiatric nurse promotes human interaction by displaying empathy, building a therapeutic relationship and establishing trust while supporting parents through the promotion of learning and growth, the provision of guidance and the supply of both material and immaterial resources.

**Structure and process of the model:** The structure of the model represents a comprehensive depiction of the conceptual relationships within the theory (Chinn et al. 2022:177). The structure and process of the model are described concurrently below.

**The context:** The context refers to the setting where the adolescent unit is situated. The context is illustrated with neutral colours. Neutrality in this study represents safety, encourages trust and a non-judgemental therapeutic environment in parents for an adolescent with a mental disorder (Olesen 2024:n.p.). A white-coloured border represents the psychiatric hospital. White symbolises purity, innocence and simplicity (Reynolds 2024:n.p.), signifying the simple and therapeutic environment that a psychiatric hospital should provide. The adolescent unit is depicted by a brown border. The colour brown is natural and earthy, representing comfort, security, protection and predictability (Cherry 2023b:n.p.). The brown colour signifies all the elements an adolescent unit should provide to the adolescent and their parents.

**The psychiatric nurse:** The psychiatric nurse is depicted by a nurse wearing a blue uniform and serves as an agent to facilitate support for parents who are caring for an adolescent with a mental disorder. Although this figure illustrates a female nurse, the model accommodates both male and female psychiatric nurses as agents. The colour blue is associated with trust and reliability (Cherry 2024:n.p.). In the context of this model, the soothing and trustworthy aura of blue helps create a comforting milieu for parents who are caring for an adolescent with a mental disorder (Shaw 2024:n.p.). The psychiatric nurse possesses the necessary skills, knowledge and attitude to facilitate support for parents and demonstrates confidence in carrying out this process. This is reflected in her open posture and the warmth in her smile. An open posture, as a form of non-verbal communication, signifies that the psychiatric nurse is welcoming, attentive and receptive to the parents (Meadus 2023:25; Stickley 2011:397).

**The parents of an adolescent:** The parents of an adolescent are represented by two figures with outstretched arms around, enclosed within a red circle. While the figure depicts two parents, the model also accommodates the recipient as a primary caregiver of the adolescent with a mental disorder, who may be biological parents (single or both) or adoptive parents. The parents who are caring for an adolescent with a mental disorder may experience grief, anxiety and a lack of professional support, with prayer as their only coping strategy. The red circle symbolises the parents’ feelings of sorrow, defeat and loss (Olesen 2024:n.p.) associated with their adolescent’s mental condition.

**Interactive dynamic process:** The relationship between the psychiatric nurse and the parents who are caring for an adolescent is an interactive dynamic process. This process is demonstrated by a blue bi-directional arrow between the psychiatric nurse and the parents who are caring for an adolescent with a mental disorder. In the case of this model, the colour blue symbolises honest communication between the psychiatric nurse and the parents, security and safety provided by the psychiatric nurse and building connections between the psychiatric nurse and the parents caring for an adolescent with a mental disorder (Rhinestones Unlimited 2023:n.p.).

**Facilitation of support:** Facilitation of support is represented by a large blue arrow on the left side of the staircase. The arrow demonstrates the dynamic and interactive nature of the facilitation of the support process. Its blue colour signifies calmness and serenity (Cherry 2024:n.p.). The colour blue reflects trustworthiness, responsibility, truth and reliability (Cherry 2024:n.p.; Van Braam 2024:n.p.). The psychiatric nurse, as the facilitator, displays reliability, calmness and confidence when facilitating the support process (Van Braam 2024:n.p.). The arrow curves over the staircase, extending from the beginning of the process in the relationship phase to its conclusion in the termination phase, and the outcome is indicative of a whole and complete perspective. The arrow is wider at the beginning of the facilitation of the support process and narrows down to a thinner arrow, symbolising the decreasing involvement of the psychiatric nurse as the facilitation of support progresses, culminating in the attainment of fresh perspectives and insights. The facilitation of support allows for a relapse through all phases of the model. In this context, the relapse implies that the process could restart at any point.

The relapse is represented by a small downward-facing red arrow next to the facilitation of support arrow. The colour red represents passion, love and desire (Cherry 2023a:n.p.). The negative connotations of red include anger, anxiety, overwhelming feelings, danger and stress (Cherry 2023a:n.p.). The downward-facing arrow represents the process of descent, self-introspection and the opportunity to start afresh.

**Facilitation of the support procedure:** The facilitation of the support process is represented by a staircase. The colour red at the base of the staircase gradually transitions to yellow at the top of the staircase as the psychiatric nurse and parents work through the facilitation process. The dark maroon-red at the bottom represents the challenges faced by the parents caring for an adolescent. Red is a powerful, strong colour that suggests strong emotions, danger, passion or aggressive behaviour (Farrow 2024a:n.p., 2024b:n.p.). Red also represents feelings of anxiety and lack of emotional control (Farrow 2024a:n.p.). A lighter shade of red represents warmth, nurturing and trustworthiness (Farrow 2024a:n.p.). As the psychiatric nurse engages in a therapeutic nurse–client relationship through the phases, the parents develop effective coping skills, become more relaxed and comfortable, are able to express their experiences without feeling judged and ultimately achieve facilitated support. The top of the staircase is yellow in colour, representing a fresh view and insight gained through the facilitation of the support process.

Cherry (2023c:n.p.) notes that being surrounded by yellow can boost happiness and positivity by giving someone a sense of inner power. The staircase is wider at the bottom, representing the parents’ challenges, that is, grief and anxiety, lack of professional support and absence of coping skills. As the parents progress through the phases, the staircase narrows, symbolising the achievement of facilitation of support. The movement of the footsteps to the next phase indicates personal growth, resilience, enlightenment and the exercise of individual choice (Hawkins 2024:n.p.). The footsteps are depicted in black in the relationship and working phases, indicating the active presence of both the psychiatric nurse and the parents in the facilitation of the support process. In the termination phase, the psychiatric nurse’s footsteps are shown in white instead of black, signifying the conclusion of their active involvement while remaining accessible, allowing the parents to restart the process if further support is needed. The facilitation of the support process involves three phases: the relationship phase, the working phase and the termination phase, which are discussed below.

**The relationship phase:** The relationship phase marks the beginning of support facilitation. In the relationship phase, the psychiatric nurse initiates the relationship phase through the use of communication skills and starts by setting an interactive dynamic process in motion to assist the parents caring for an adolescent with a mental disorder in facilitating support. The relationship phase consists of three components: establishing trust, building a therapeutic relationship and demonstrating empathy. The psychiatric nurse focuses on building a trusting and therapeutic connection with the parents. This is achieved by obtaining informed consent, establishing rapport and setting clear, professional boundaries. The nurse employs active listening, demonstrates empathy and validates the parents’ feelings to create a secure, non-judgmental environment. Through consistent, respectful and honest communication, the nurse fosters an atmosphere of mutual respect where parents feel understood and comfortable sharing their experiences. The psychiatric nurse and the parents will collaborate to establish objectives for their forthcoming sessions.

**The working phase:** During the working phase, the psychiatric nurse supports the parents by promoting learning and growth, providing guidance and mobilising both material and immaterial resources.

Learning and growth are facilitated through the encouragement of collaboration and shared decision-making with the parents. The psychiatric nurse ensures that the physical environment is conducive to the promotion of learning and growth and empowers and encourages parents to actively participate, recognising their role in their personal learning and growth. The psychiatric nurse creates a supportive and positive environment that encourages parents to reflect on their experiences, explore thoughts, emotions and behaviours and strengthen coping skills. Regarding guidance provision, the psychiatric nurse facilitates this through the involvement and participation of parents in decision-making and problem-solving. The psychiatric nurse encourages parents to identify and reflect on personal and caregiving goals, collaboratively developing strategies to achieve them.

This process empowers parents to enhance their coping, problem-solving and decision-making skills. The psychiatric nurse maintains a non-judgmental attitude and creates a safe space in which parents can express their concerns and share their experiences. The psychiatric nurse continues to develop their skills, knowledge and expertise to provide guidance and reassurance to parents. The psychiatric nurse will mobilise material and immaterial resources, which include family therapy, individual therapy, group therapy, mental health education and referrals. The parents will develop coping skills that will enhance their quality of life. The psychiatric nurse will use therapeutic communication skills to manage the facilitative process effectively.

**The termination phase:** The final phase is the termination phase, in which the psychiatric nurse prepares for the conclusion of the process and evaluates the achievement of facilitation of support. The termination phase enables the parents caring for an adolescent to reflect on the work undertaken across all phases and to evaluate facilitated support. This phase marks the conclusion of the relationship between the psychiatric nurse and the parents. During this phase, the parents provide feedback on the facilitation of support and reflect on the skills they have acquired in the process. The parents need to ensure that the goals established at the outset have been achieved.

**The outcome:** The outcome of the process is facilitated support for parents caring for an adolescent with a mental disorder. When these parents engage in the process of facilitation of support with the psychiatric nurse, the expected outcome is facilitated support for parents caring for an adolescent with a mental disorder, and it is evidenced by parents displaying healthy coping and problem-solving skills, improved support-seeking abilities and the capacity to manage the difficulties of everyday life. Parents caring for an adolescent with a mental disorder who have received support are represented by two parents and an adolescent holding hands, smiling and demonstrating happiness as a result of the facilitation of the support process.

## Evaluation of the model

The model was evaluated according to the criteria outlined by Chinn et al. (2022:169–176). Five questions guided critical reflection, namely: How clear, simple, general, accessible and important is the model? The model was evaluated by a panel of seven experts experienced in research theory, nursing education, nursing research and advanced child and adult psychiatric nursing.

The panel of experts who evaluated the model consisted of one full-time professor, two senior lecturers with PhDs, one advanced psychiatric nurse with a PhD qualification and two child psychiatric nurses with advanced university diplomas in child psychiatric nursing. The panel of experts all had between 8 and 28 years of experience in academic and clinical settings. The model was found to meet the criteria for model development, which are discussed below.

### Clarity

Chinn et al. (2022:171) state that structural clarity, structural consistency, semantic clarity and semantic consistency determine the clarity of the model.

#### Semantic clarity and consistency

Semantic clarity and consistency refer to clarifying the intended meaning of the concepts within their theoretical framework (Chinn et al. 2022:171–173). The following comments were made:

‘The model is concise, informative and easy to apply.’ (Participant 5, Child Psychiatric Nurse, 11 years’ experience)

‘The model is clear and easy to follow.’ (Participant 2, Professor, 10 years’ experience)

#### Structural clarity and consistency

Structural clarity and consistency refer to understanding how concepts connect within the theory and how the entire theory integrates (Chinn et al. 2022:171–173). The following comments were made:

‘Structure and phases are clear.’ (Participant 6, Child Psychiatric Nurse, 20 years’ experience)

‘The model is clear.’ (Participant 4, Senior Lecturer, 9 years’ experience)

### Simplicity

The model’s simplicity refers to a few concepts within each category and the clear connections between them (Chinn et al. 2022:174). The researcher ensured that the concepts were simple and used a limited number of key concepts. The evaluators stated that the model’s concepts were simple. The comments were as follows:

‘The model is simplistic, easy to follow and easy to apply.’ (Participant 3, Advanced Psychiatric Nurse, 28 years’ experience)

‘Three-phase structure is simple.’ (Participant 6, Child Psychiatric Nurse, 20 years’ experience)

### Generality

The model’s generality refers to its transferability to different settings and its purpose; a model’s breadth of scope should allow it to be used in other situations (Chinn et al. 2022:174). The evaluators indicated the model could be used in different settings. Their comments are reflected as follows.

‘The model could be generalised to other parents or families who have children with other challenging conditions.’ (Participant 1, Senior Lecturer, 8 years’ experience)

‘The model speaks to a specific target group with special and specific needs and can benefit parents and, in the end, the adolescent, even in different settings. The outline also speaks to the generalisability of the model.’ (Participant 3, Advanced Psychiatric Nurse, 28 years’ experience)

### Accessibility

The model’s accessibility addresses the extent to which empirical measures for the concepts can be identified and the feasibility of attaining the model’s purpose (Chinn et al. 2022:175). The evaluators found the model to be accessible. Some of their comments are reflected as follows.

‘The model is written in a way that is accessible to healthcare professionals. The language is clear and the structure is easy to follow.’ (Participant 6, Child Psychiatric Nurse, 20 years’ experience)

‘The model can be used by other departments that have parents experiencing their children with different mental and medical conditions.’ (Participant 1, Senior Lecturer, 8 years’ experience)

### Importance

The model’s importance refers to the extent to which it contributes to improved nursing practice, education and research (Chinn et al. 2022:176). The model must prove clinically beneficial, expand current knowledge, provide a basis for psychiatric nursing practice and consistently improve patient outcomes in different contexts (Chinn et al. 2022:176).

‘The model addresses a critical issue of supporting parents caring for adolescents with mental disorders. Focuses on establishing a therapeutic relationship, promoting learning and growth. Empowering parents is essential for improving adolescent mental health outcomes and family functioning.’ (Participant 6, Child Psychiatric Nurse, 20 years’ experience)

‘This model is important as parents are the primary caregivers of adolescents; they, too, experience high levels of stress, uncertainty, and emotional distress. A supportive space for caregivers leads to better family dynamics and a more stable home environment.’ (Participant 4, Senior Lecturer, 9 years’ experience)

## Limitations

This study does not address the implementation of the model or the evaluation of its implementation.

## Recommendations

Recommendations for nursing practice include that psychiatric nurses could use the model to support parents who are caring for an adolescent with a mental disorder. The model has the potential to empower psychiatric nurses, enhancing their confidence in their skills and expanding their mental health knowledge. It may also strengthen the relationship between the psychiatric nurse and the parents caring for an adolescent with a mental disorder. It is recommended that mental health institutions should adopt policies promoting the implementation of formalised parental support programmes led by psychiatric nurses. This will equip parents with the knowledge and skills, as well as strengthen the nurses’ competencies. For nursing research, it is recommended that the model be used in further research to explore the experiences of psychiatric nurses in applying it and to evaluate the effectiveness of facilitation of support models for parents. Recommendations for nursing education include incorporating the model into undergraduate and postgraduate training for psychiatric nursing students. This could be beneficial to assist psychiatric nursing students to be better equipped to facilitate support for parents caring for an adolescent with a mental disorder.

## Conclusion

The purpose of this article was to describe and evaluate a model for psychiatric nurses to facilitate support for parents who are caring for an adolescent with a mental disorder. The model was described using the method of Chinn et al. (2022). The structure and process of the model were described. The model evaluation was described using the five relevant questions for critical reflection. Ethical considerations and measures to ensure trustworthiness were described. The article addresses a gap by developing, describing and evaluating a model for psychiatric nurses to facilitate support for parents caring for an adolescent with a mental disorder. It provides recommendations for nursing practice, nursing research and nursing education. Limitations were also discussed.
